# *Bryophryne phuyuhampatu* sp. n., a new species of Cusco Andes frog from the cloud forest of the eastern slopes of the Peruvian Andes (Amphibia, Anura, Craugastoridae)

**DOI:** 10.3897/zookeys.685.12152

**Published:** 2017-07-13

**Authors:** Alessandro Catenazzi, Alex Ttito, M. Isabel Diaz, Alexander Shepack

**Affiliations:** 1 Department of Zoology, Southern Illinois University, Carbondale, USA; 2 Centro de Ornitología y Biodiversidad, Lima, Perú; 3 Museo de Historia Natural de la Universidad Nacional de San Antonio Abad del Cusco, Plaza de armas s/n (Paraninfo universitario), Cusco, Perú; 4 Museo de Biodiversidad del Perú, Urbanización Mariscal Gamarra A-61, Zona 2, Cusco, Perú; Alessandro Catenazzi

**Keywords:** leaf litter amphibian, montane forest, Paucartambo, taxonomy, anfibio de hojarasca, Paucartambo, bosque nublado, taxonomía

## Abstract

A new species of *Bryophryne* from the humid montane forest of the Department of Cusco, Peru, is described. Specimens were collected at 2795–2850 m a.s.l. in the Área de Conservación Privada Ukumari Llaqta, Quispillomayo valley, in the province of Paucartambo. The new species is readily distinguished from all other species of *Bryophryne* by having green coloration on dorsum, and blue flecks on flanks and ventral parts. Specimens are characterized by lacking a distinct tympanic annulus, tympanic membrane, and dentigerous processes of vomers, and by having dorsal skin shagreen, discontinuous dorsolateral folds, skin tuberculate on flanks, skin areolate on ventral surfaces of the body, and fingers and toes without lateral fringes or webbing. The new species has a snout–vent length of 14.2–16.9 mm in three males and 22.2–22.6 mm in two females, and is smaller than all other congeneric species except for *B. abramalagae*. Generic allocation is supported by low genetic distances of the 16S mitochondrial gene and morphological similarity with other species of *Bryophryne*, and geographic distribution. *Bryophryne phuyuhampatu* **sp. n.** is only known from the type locality, a cloud forest along the Quispillomayo River in the upper Nusiniscato watershed.

## Abstract (es)

Se describe una nueva especie de *Bryophryne* de los bosques nublados del Departamento de Cusco en Perú. Los especímenes fueron colectados a una elevación de 2795–2850 m en el Área de Conservación Privada Ukumari Llaqta, valle del río Quispillomayo, provincia de Paucartambo. La nueva especie se diferencia fácilmente de todas las demás especies de *Bryophryne* por tener coloración verde en la espalda y manchitas azuladas en los flancos y las superficies ventrales. Los especímenes se caracterizan por carecer de procesos vomerianos y de anillo y membrana timpánicos, y por poseer piel dorsal finamente granulada, pliegues dorsolaterales discontinuos, piel en los flancos verrugosa, y cubierta de verrugas en areola en las partes ventrales, y por carecer de membrana basal y quillas laterales en los dedos. La nueva especie tiene una longitud hocico–cloaca (LHC) de 14.2–16.9 mm en tres machos y de 22.2–22.6 mm en dos hembras, siendo menor que todas las demás especies de *Bryophryne* excepto por *B. abramalagae*. Distancias genéticas bajas para el gen mitocondrial 16S, similitud morfológica con otras especies de *Bryophryne* y distribución geográfica indican que la nueva especie forma parte del género *Bryophryne*. *Bryophryne phuyuhampatu* **sp. n.** se conoce únicamente de su localidad tipo, un bosque nublado en el valle del torrente Quispillomayo, en la parte alta de la cuenca del río Nusiniscato.

## Introduction

The frog genus *Bryophryne* currently includes nine species, all endemic to the southern Peruvian Department of Cusco, and distributed across the humid highland grasslands and forests from 2350 to 4000 m a.s.l. in the Amazonian slopes of the Andes (Chaparro et al. 2015; Duellman and Lehr 2009; Frost 2017; Padial et al. 2014). Molecular phylogenies support placement of the genus within the Holoadeninae in the family Craugastoridae (Hedges et al. 2008; Padial et al. 2014). High-Andean genera of Holoadeninae are characterized by having narrow terminal digits on the fingers and toes and by lacking circumferential grooves, but are generally indistinguishable on the basis of morphological traits (Duellman and Lehr 2009; Hedges et al. 2008).

Knowledge of the diversity of this genus has improved dramatically over the past decade (Chaparro et al. 2015), contributing to Peru's high rate of new species discoveries (Catenazzi 2015). Whereas only the type species of the genus, *B. cophites* (Lynch), was known until 2007, all other congeneric species have been discovered since 2007 (Chaparro et al. 2007; Chaparro et al. 2015; Lehr and Catenazzi 2008; Lehr and Catenazzi 2009a; Lehr and Catenazzi 2010). These recent discoveries confirm that species of *Bryophryne* are predominantly mountaintop species, and that each mountain pass is occupied by up to three different species of seemingly restricted geographic distribution (Lehr and Catenazzi 2009a; Lehr and Catenazzi 2010). Mountain passes as close as 50 km share no species of *Bryophryne*, suggesting high levels of beta diversity.

During May and June of 2015 and 2016 we explored two valleys of the eastern side of the Cordillera de Paucartambo within the Área de Conservación Privada Ukumari Llaqta (Catenazzi and Ttito 2016), a protected area recognized by a Peruvian environmental ministerial decree in 2011. This private area is owned and managed by local communities, whose members permitted our work and guided us through the high-elevation grasslands, montane scrub, and down to the higher reaches of the humid montane forest. Our work in the Japumayo Valley in 2015 led to the discovery of *Psychrophrynella chirihampatu* (Catenazzi and Ttito 2016). In 2016 we surveyed the adjacent Quispillomayo Valley, where we found specimens of a new species of *Bryophryne* in the humid montane forest. Here we report on these recent surveys, and describe the new species.

## Materials and methods

The format of the diagnosis and description follows Duellman and Lehr (2009) and Lynch and Duellman (1997), except that the term dentigerous processes of vomers is used instead of vomerine odontophores (Duellman et al. 2006). We follow Hedges et al. (2008) for taxonomy, except for family placement (Pyron and Wiens 2011). We derived meristic traits of similar species from specimens examined, published photographs, or species descriptions.

Specimens were fixed and preserved in 70% ethanol. Sex and maturity of specimens were determined by observing sexual characters and gonads through dissections. We measured the following variables (Table 1) to the nearest 0.1 mm with digital calipers under a stereomicroscope:

**SVL** snout–vent length

**TL** tibia length

**FL** foot length (distance from proximal margin of inner metatarsal tubercle to tip of Toe IV)

**HL** head length (from angle of jaw to tip of snout)

**HW** head width (at level of angle of jaw)

**ED** eye diameter

**TY** tympanum diameter

**IOD** interorbital distance

**EW** upper eyelid width

**IND** internarial distance

**E–N** eye–nostril distance (straight line distance between anterior corner of orbit and posterior margin of external nares)

**Table 1.** Genetic distances (uncorrected p-distances) estimated from the non-coding 16S rRNA mitochondrial fragment (GenBank accession codes in parentheses) between *Bryophryne phuyuhampatu* and related taxa (in boldface the most closely related species) of the subfamily Holoadeninae (Craugastoridae).

	*Barycholos pulcher* (EU186709)	*Bryophryne bakersfield* (KT276289)	*Br. bustamantei* (KT276293)	*Br. cophites* (EF493537)	*Br. phuyuhampatu* CORBIDI 18224 (MF419254)	*Br. phuyuhampatu* MUBI 14654 (MF419259)	*Br. phuyuhampatu* CORBIDI 18225 (MF419255)	*Br. phuyuhampatu* CORBIDI 18226 (MF419256)	*Br. phuyuhampatu* CORBIDI 18227 (MF419257)	*Br. phuyuhampatu* CORBIDI 18228 (MF419258)	*Br. phuyuhampatu* MUBI 14655 (MF419260)	*Holoaden luederwaldti* (EU186710)	*Noblella lochites* (EU186699)	*Psychrophrynella chirihampatu* (KU884559)	*P. guillei* (AY843720)	*P. usurpator* (EF493714)	*P. wettsteini* (EU186696)
*Barycholos pulcher* (EU186709)																	
*Bryophryne bakersfield* (KT276289)	0.19																
*Br. bustamantei* (KT276293)	0.26	0.05															
*Br. cophites* (EF493537)	0.24	0.04	0.15														
*Br. phuyuhampatu* CORBIDI 18224 (MF419254)	0.19	**0.04**	**0.06**	**0.06**													
*Br. phuyuhampatu* MUBI 14654 (MF419259)	0.20	**0.04**	**0.06**	**0.06**	0.00												
*Br. phuyuhampatu* CORBIDI 18225 (MF419255)	0.20	**0.04**	**0.07**	**0.06**	0.00	0.00											
*Br. phuyuhampatu* CORBIDI 18226 (MF419256)	0.20	**0.04**	**0.06**	**0.06**	0.00	0.00	0.00										
*Br. phuyuhampatu* CORBIDI 18227 (MF419257)	0.20	**0.04**	**0.06**	**0.06**	0.00	0.00	0.00	0.00									
*Br. phuyuhampatu* CORBIDI 18228 (MF419258)	0.20	**0.04**	**0.06**	**0.06**	0.00	0.00	0.00	0.00	0.00								
*Br. phuyuhampatu* MUBI 14655 (MF419260)	0.21	**0.04**	**0.06**	**0.06**	0.00	0.00	0.00	0.00	0.00	0.00							
*Holoaden luederwaldti* (EU186710)	0.27	0.18	0.23	0.21	0.17	0.17	0.17	0.19	0.19	0.19	0.20						
*Noblella lochites* (EU186699)	0.25	0.20	0.26	0.24	0.20	0.19	0.20	0.18	0.19	0.18	0.19	0.23					
*Psychrophrynella chirihampatu* (KU884559)	0.21	0.19	0.19	0.19	0.17	0.17	0.18	0.18	0.18	0.18	0.17	0.17	0.21				
*P. guillei* (AY843720)	0.24	0.14	0.12	0.22	0.13	0.12	0.14	0.13	0.13	0.13	0.13	0.23	0.27	0.15			
*P. usurpator* (EF493714)	0.29	0.20	0.24	0.24	0.20	0.19	0.20	0.20	0.20	0.20	0.20	0.22	0.27	0.05	0.21		
*P. wettsteini* (EU186696)	0.26	0.17	0.20	0.21	0.16	0.15	0.15	0.15	0.15	0.15	0.15	0.21	0.24	0.17	0.13	0.21	

Hand length was measured as the distance from the proximal margin of thenar tubercle to tip of Finger III. Fingers and toes are numbered preaxially to postaxially from I–IV and I–V respectively. We determined comparative lengths of toes III and V by adpressing both toes against Toe IV; lengths of fingers I and II were determined by adpressing these fingers against each other. In two female specimens, the ovaries were dissected, the eggs extracted, and their diameter measured under a stereomicroscope to the nearest 0.01 with a digital caliper.

**Figure 1.**
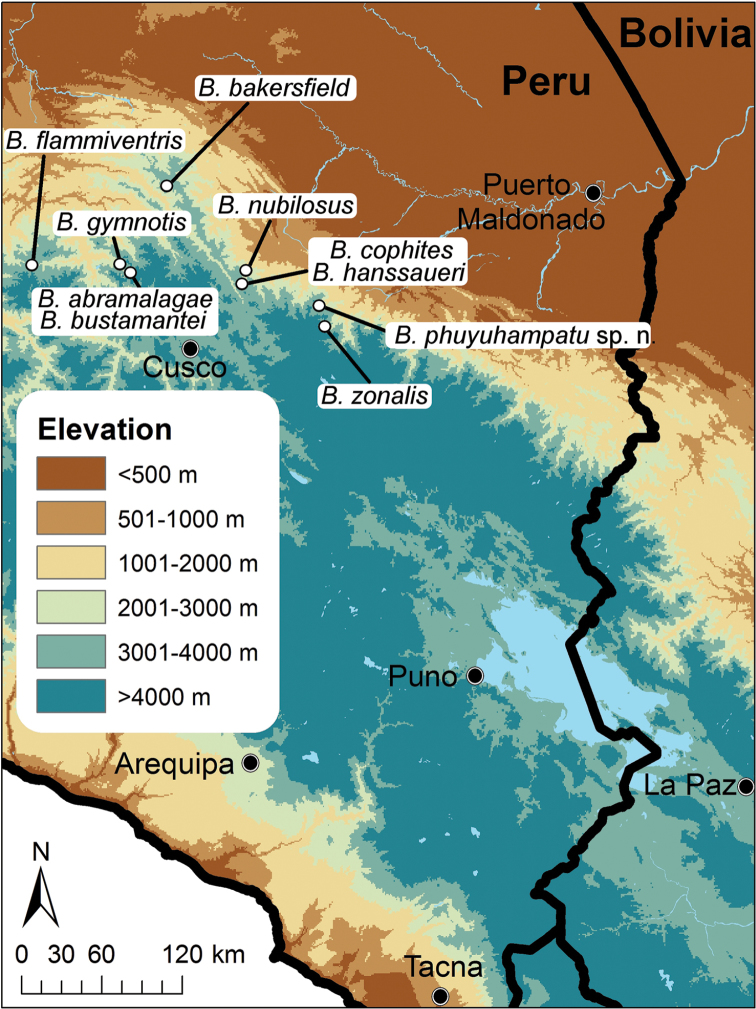
Map of Peru indicating the type localities of species of *Bryophryne*.

Standard protocols were used to extract, amplify and sequence the non-coding 16S rRNA mitochondrial fragment (Catenazzi and Ttito 2016), and new sequences were deposited in GenBank (Table 1). Variation in coloration was described on the basis of field notes and photographs of live frogs. Photographs taken by A. Catenazzi of live specimens, including types and non-collected specimens, and of preserved types have been deposited at the Calphoto online database (http://calphotos.berkeley.edu). Locality names follow the spelling of the Carta Nacional “Chontachaca” (27-t), Instituto Geográfico Nacional, Lima. Elevation data for the map (Figure 1) were obtained from http://www.diva-gis.org.

Specimens examined are listed in Appendix I; codes of collections are:

**CORBIDI** Herpetology Collection, Centro de Ornitología y Biodiversidad, Lima, Peru

**MUBI** Museo de Biodiversidad del Perú, Cusco

**KU** Natural History Museum, The University of Kansas, Lawrence, Kansas, USA

**MUSM** Museo de Historia Natural Universidad Nacional Mayor de San Marcos, Lima, Peru

**MHNG** Muséum d'Histoire Naturelle, Genève, Switzerland

Research was approved by Institutional Animal Care and Use Committees of Southern Illinois University Carbondale (protocol #13–027). The permit to carry on this research has been issued by the Peruvian Ministry of Agriculture (permit #292–2014-MINAGRI-DGFFS-DGEFFS). The Comunidad Campesina Japu Q'eros authorized our work on their land.

### Bryophryne phuyuhampatu sp. n.

Taxon classificationAnimaliaAnuraCraugastoridae

http://zoobank.org/BB13FD82-3470-4E31-A6EF-87606B0CC356

#### Holotype.

**Figure 2.**
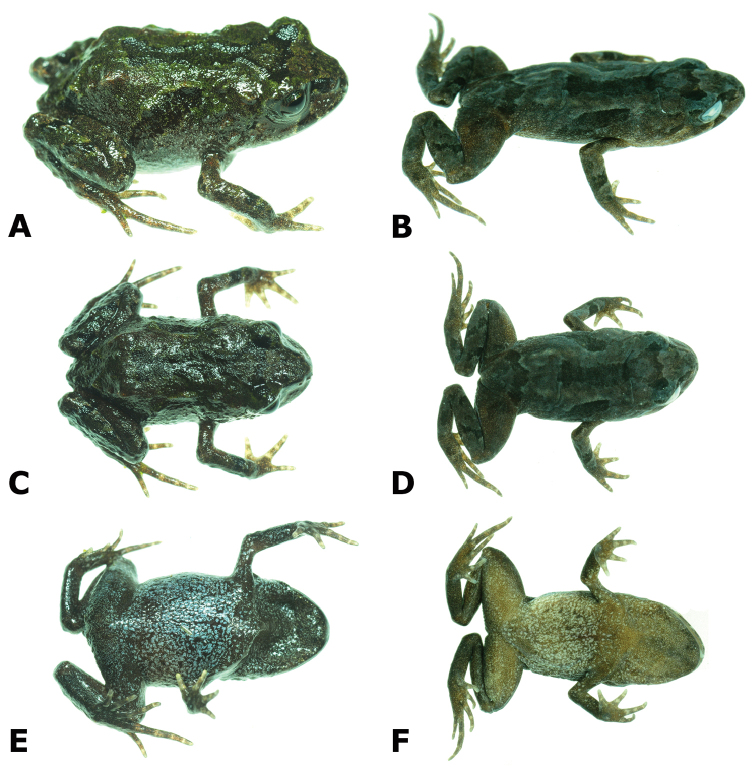
Live (left column) and preserved (right column) specimen of the holotype of *Bryophryne phuyuhampatu* sp. n., male CORBIDI 18226 (SVL 16.9 mm) in dorsolateral **A, B** dorsal **C, D** and ventral **E, F** views. Photographs by A. Catenazzi.

**Figure 3.**
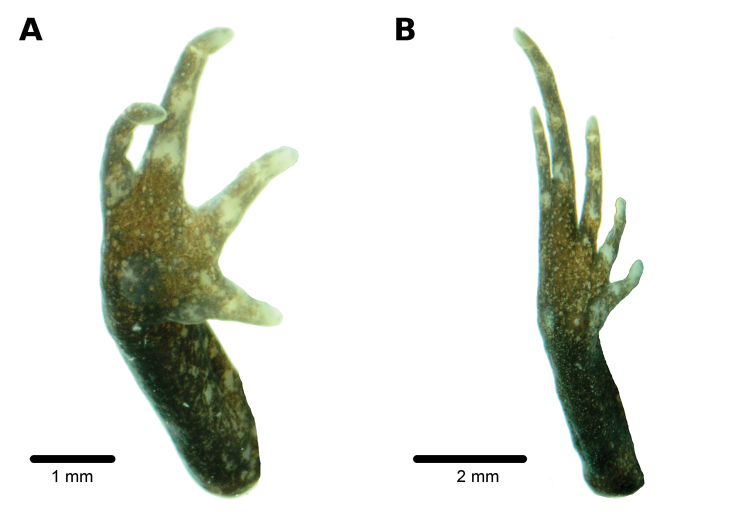
Ventral views of hand **A** and foot **B** of holotype, CORBIDI 18226 (hand length 4.1 mm, foot length 6.6 mm) of *Bryophryne phuyuhampatu* sp. n. Photographs by A. Catenazzi.

(Figs 1–3, Table 2). CORBIDI 18226, an adult male (Figs 2, 3) from 13°22'12.14''S; 71°6'49.82''W (WGS84), 2795–2850 m a.s.l., Quispillomayo valley, Área de Conservación Privada (ACP) Ukumari Llaqta, Distrito Paucartambo, Provincia Paucartambo, Departamento de Cusco, Peru, collected by A. Catenazzi, A. Shepack, M. I. Diaz and A. Ttito on 27 May 2016.

#### Paratopotypes.

**Figure 4.**
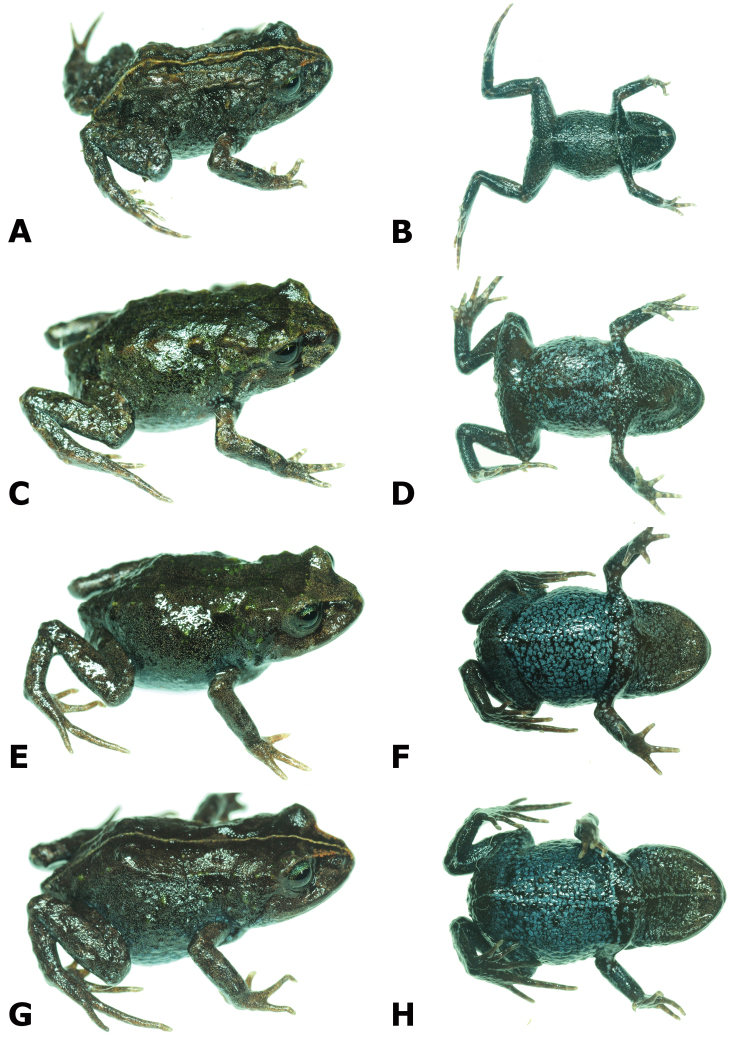
Dorsolateral and ventral views of four paratypes of *Bryophryne phuyuhampatu* sp. n. showing variation in dorsal and ventral coloration. Male CORBIDI 18225, SVL = 14.2 mm **A, B** Male MUBI 14655, SVL = 15.9 mm **C, D** Female CORBIDI 18224, SVL = 22.6 mm **E, F**. Female MUBI 14654, SVL = 22.2 mm **G, H** Photographs by A. Catenazzi.

(Fig. 4, Table 2). Four specimens: two females, CORBIDI 18224 and MUBI 14654, and one male, CORBIDI 18225, collected with the holotype on 27 May 2016; and one male, MUBI 14655 collected on 28 May 2016.

**Table 2.** Range and average (± standard deviation) measurements (in mm) of type series of *Bryophryne phuyuhampatu* sp. n.

Characters	Females (n = 2)	Males (n = 3)
SVL	22.2–22.6	14.2–16.9 (15.7 ± 0.8)
TL	8.1–8.4	5.6–6.5 (6.1 ± 0.3)
FL	9.2–9.9	5.8–6.6 (6.3 ± 0.3)
HL	8.1–8.9	4.8–5.5 (5.1 ± 0.2)
HW	7.0–7.6	4.4–5.4 (5.0 ± 0.3)
ED	2.1	1.6–1.8 (1.7 ± 0.1)
IOD	3.6–3.7	2.2–2.6 (2.4 ± 0.1)
EW	1.4–1.6	1.0–1.1 (1.1 ± 0.0)
IND	2.0–2.1	1.4–1.5 (1.5 ± 0.0)
E–N	1.5–2.0	1.3 (1.3 ± 0.0)
TL/SVL	0.36–0.38	0.38–0.39
FL/SVL	0.41–0.45	0.39–0.41
HL/SVL	0.36–0.40	0.31–0.34
HW/SVL	0.31–0.34	0.31–0.33
HW/HL	0.85–0.86	0.92–1.06
E–N/ED	0.71–0.95	0.72–0.81
EW/IOD	0.38–0.44	0.38–0.50

#### Referred specimens.

Three juveniles, CORBIDI 18227, 18228, and MUBI 14665, collected with the holotype and paratopotypes on 27 May 2016.

#### Generic placement.

A new species of *Bryophryne* as defined by Duellman and Lehr (2009), Hedges et al. (2008), and Padial et al. (2014). Frogs of the genus *Bryophryne* are morphologically similar and closely related to *Barycholos*, *Holoaden*, *Noblella* and *Psychrophrynella* (Chaparro et al. 2015; Hedges et al. 2008; Heinicke et al. 2007; Padial et al. 2014). Genetic data confirm generic placement of the new species within *Bryophryne* (Table 1). We found substantial genetic distances (uncorrected p-distances from 3.7–6.7%; Table 1) between *B. phuyuhampatu* and congeneric species for which mitochondrial sequence data were available (*B. bakersfield*, *B. bustamantei*, and *B. cophites*). The most closely related species is *B. bakersfield* (16S uncorrected p-distance: 3.7–4.1%), followed by *B. cophites* (5.6–6.2%) and *B. bustamantei* (5.6–6.7%). Regarding species from other genera, *B. phuyuhampatu* had genetic distances ranging from 12.4% (*Psychrophrynella guillei*) to 20.8% (*Barycholos pulcher*). In addition to the molecular data, the new species is assigned to *Bryophryne* rather than any of the other genera on the basis of overall morphological resemblance with the type species *B. cophites*, including head narrower than body, short limbs, and tympanic membrane and annulus usually absent (absent in most species of *Bryophryne*, except for *B. flammiventris* and *B. gymnotis*), and geographic distribution within the Departamento Cusco, where all other species of *Bryophryne* occur.

#### Diagnosis.

A new species of *Bryophryne* characterized by: (1) skin on dorsum shagreen; skin on venter areolate, discoidal fold absent, thoracic fold present; dorsolateral folds irregular and discontinuous; (2) tympanic membrane and tympanic annulus absent; (3) snout rounded in dorsal view and in profile; (4) upper eyelid with two small tubercles, narrower than IOD; cranial crests absent; (5) dentigerous process of vomers absent; (6) vocal sac and slits absent; nuptial pads absent; (7) Finger I much shorter than Finger II; tips of digits slightly pointed; (8) fingers lacking lateral fringes; (9) outer edge of forearm bearing small tubercles; (10) heel bearing minute tubercles; inner tarsal fold absent; outer edge of tarsus bearing small tubercles; (11) inner metatarsal tubercle prominent, ovoid, of similar relief and slightly larger than ovoid, outer metatarsal tubercle; supernumerary plantar tubercles indistinct; (12) toes lacking lateral fringes; webbing absent; toes III and V about equal in length; tips of digits slightly pointed; (13) in life, dorsum tan to green and brown with dark brown markings, greenish blue on lower flanks; some specimens with a yellow middorsal line extending from tip of snout to cloaca and to the posterior surface of thighs; interorbital bar present; chest, belly and ventral parts of forearms and legs dark brown with grayish blue flecks; throat brown with flecks turning from gray-blue to copper near tip of mouth; palmar and plantar surfaces brown with lighter fingers and toes; (14) SVL 14.2–16.9 in males (n = 3), 22.2–22.6 in females (n = 2).

#### Comparisons.

The new species differs from other members of the genus by having green coloration on dorsum and blue coloration on flanks and ventral parts. Furthermore, *B. phuyuhampatu* differs from other species by the following combination of characters (condition for comparing species in parenthesis): from *B. abramalage* by having proportionally longer feet with FL/SVL from 0.41–0.45 (0.37–0.42), narrower head with HW/HL from 0.85–0.86 (0.97–1.07), and inner metatarsal tubercle larger than outer metatarsal (inner half the size of outer metatarsal tubercle); *B. flammiventris* and *B. gymnotis* by lacking a tympanum (present), from *B. bakersfield* and *B. bustamantei* by having discontinuous dorsolateral folds (continuous), from *B. cophites* by females being much smaller (22.6 mm vs. 35.8 mm), from *B. hanssaueri* by lacking bright orange coloration on throat (present), from *B. nubilosus* by having toes III and V similar in length (toe V > III), and from *B. zonalis* by having blue-gray mottled coloration on belly (distinctive black mottled coloration, variably confined to lower portion of belly). The new species further differs from *B. gymnotis* by having vomers lacking dentigerous processes (present), from *B. cophites* by males lacking nuptial pads (present), and from *B. bakersfield*, *B. bustamantei*, *B. flammiventris* and *B. gymnotis* for males lacking vocal slits (present).

*Bryophryne phuyuhampatu* (max. SVL 22.6 mm) is much smaller than *B. bakersfield* (31.1 mm), *B. cophites* (35.8 mm; pers. obs.), *B. hanssaueri* (29.3 mm; pers. obs.), and *B. zonalis* (32.4 mm), smaller than *B. bustamantei* (23.4 mm), *B. flammiventris* (24.1), *B. hanssaueri* (24.6 mm), and about the same size of *B. abramalagae* (20.1 mm) and *B. nubilosus* (26.0 mm; pers. obs.). Five other small species of craugastorid frogs of the subfamily Holoadeninae are known to occur in montane forests and high Andean grasslands south of the Apurimac canyon in Peru: *Noblella madreselva*, *N. pygmaea*, *Psychrophrynella bagrecito*, *P. chirihampatu* and *P. usurpator*, which all possess a visible tympanic annulus.

#### Description of holotype.

Adult male (16.9 mm SVL); head narrower than body, its length 33% of SVL; head wider than long, head length 83% of head width; head width 32% of SVL; snout short, rounded in dorsal and lateral views (Fig. 2), eye diameter 35% of head length, its diameter 1.2 times as large as its distance from the nostril; nostrils slightly protuberant, close to snout, directed dorsolaterally; canthus rostralis slightly straight in dorsal view, rounded in profile; loreal region slightly concave; lips rounded; upper eyelids with two small tubercles; upper eyelid width 46% of interorbital distance; interorbital region flat, lacking cranial crests; eye-nostril distance 81% of eye diameter; supratympanic fold short and weak; tympanic membrane and tympanic annulus absent; postrictal tubercles absent. Vocal sac and vocal slits absent. Choanae ovoid, small, positioned far anteriorly and laterally, widely separated from each other; dentigerous processes of vomer and vomerine teeth absent; tongue large, ovoid, about 2.5 times as long as wide, not notched posteriorly.

Skin on dorsum shagreen with small, scattered tubercles; dorsolateral folds discontinuous, extending from posterior margin of upper eyelid to sacral region; skin on flanks tuberculate; skin on throat smooth, skin on chest, and belly areolate; thoracic fold present, discoidal fold absent; cloaca slightly protuberant, cloacal sheath short, cloacal region without tubercles. Outer surface of forearm with minute tubercles; palmar tubercle flat and oval, approximately same length but twice the width of elongate, thenar tubercle; few supernumerary tubercles low, ovoid; subarticular tubercles prominent, ovoid in ventral view, rounded in lateral view, largest at base of fingers; fingers lacking lateral fringes; Finger I much shorter than Finger II; relative lengths of fingers 3 > 4 = 2 > 1 (Fig. 3); tips of digits slightly pointed, lacking circumferential grooves (Fig. 3); forearm lacking tubercles.

Hindlimbs short and robust, tibia length 38% of SVL; foot length 39% of SVL; upper surfaces of hindlimbs shagreen with scattered, minute tubercles; posterior surface of thighs tuberculate to areolate, ventral surface areolate; heel with minute tubercles (not visible in preservative); inner edge of tarsus without tubercles, outer edge of tarsus with small tubercles; inner metatarsal tubercle prominent, ovoid, of similar relief and slightly larger than ovoid, outer metatarsal tubercle; supernumerary plantar tubercles indistinct; subarticular tubercles low, ovoid in dorsal view; toes lacking lateral fringes, not webbed; toe tips weakly pointed, not expanded laterally, about as large as those on fingers; relative lengths of toes: 4 > 3 = 5 > 2 > 1 (Fig. 3); foot length 32% of SVL.

Measurements of holotype (all in mm): SVL 16.9, TL 6.5, FL 6.6, HL 5.5, HW 5.4, ED 1.6, IOD 2.4, EW 1.1, IND 1.5, E–N 1.3.

#### Coloration of holotype in life.

(Fig. 2). Dorsum green and brown with a dark brown marking extending from the interorbital bar to a mid-dorsal longitudinal band, a horizontal dark mark near the sacral region, and an oblique dark band on each flank. Dorsal surfaces of arms and legs dark brown, with transverse dark bars on forearms and hind limbs. Lower flanks with greenish blue flecks. Chest, belly and ventral parts of forearms and legs dark brown with grayish blue flecks. Iris grayish blue with a medial copper band. Throat brown with flecks turning from gray-blue to copper near the tip of the mouth. Palmar and plantar surfaces brown; tips of fingers and toes light brown to yellow.

#### Coloration of holotype in alcohol.

(Fig. 2). Similar to coloration in life, but dorsal surfaces grayish tan with higher contrast of dorsal markings. Ventral surfaces beige to brown with cream flecks.

#### Variation.

Coloration in life is based on field notes and photographs taken by A. Catenazzi of the paratopotypes (Fig. 4; photographs available through Calphoto database). The amount of dorsal green coloration varies among specimens. While juvenile MUBI 14665 and male MUBI 14655 are similar to the holotype in having a generally greenish dorsum, all other specimens have dark tan to brown dorsum, with just a few tubercles colored green. Female MUBI 14654, male CORBIDI 18225 and juvenile CORBIDI 18228 have a yellow middorsal line extending from the tip of the snout to the cloaca and to the posterior surface of the thighs.

The summary of measurements of all types is reported in Table 2.

#### Etymology.

The specific name *phuyuhampatu* is a combination of Quechua words used in apposition meaning “toad” (“hampa'tu”) that lives in the “fog” (“phuyu”).

#### Distribution, natural history, and threats.

**Figure 5.**
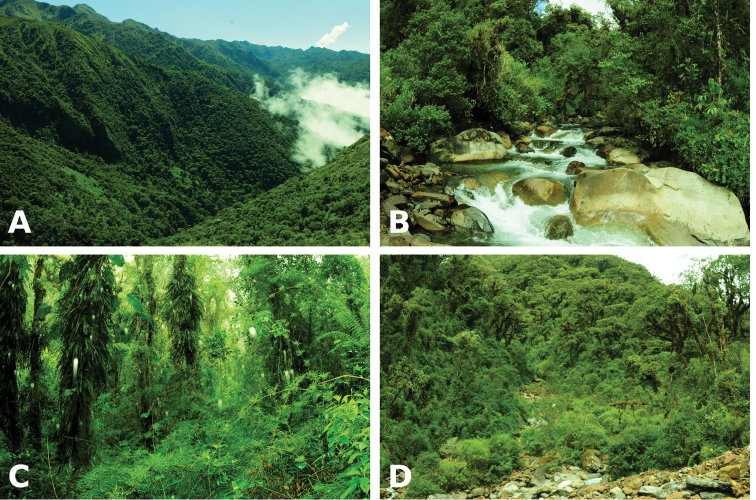
Collection localities of *Bryophryne phuyuhampatu* sp. n. in the upper Quispillomayo River valley **A** lookout from 3050 m a.s.l.: frogs were found under mosses, leaves and rocks in the cloud forest along the Quispillomayo River **B** such as the type locality at 2850 m a.s.l. **C** and disturbed forest at the confluence with a stream at 2795 m a.s.l. **D** Photographs by A. Catenazzi.

*Bryophryne phuyuhampatu* was discovered during a rapid amphibian survey in the upper Quispillomayo Valley (Fig. 5A) from 22 to 31 May 2016. The Quispillomayo torrent (Fig. 5B) is a tributary of the Nusiniscato River, which reaches the Araza River downstream of Quincemil, in the upper Madre de Dios drainage. During the inventory high-Andean grasslands (puna; 3350–4515 m a.s.l.), a forest patch of tasta (*Escallonia myrtillioides*), kishuar (*Buddleja incana*) and qeñua (*Polylepis incana*) at 4280 m a.s.l., montane scrub, disturbed areas and other transitional formations along the treeline around 3350 m a.s.l., and the montane cloud forest from 2780–3350 m a.s.l. were sampled. Frogs were searched for under rocks, logs, mosses, and in the leaf litter and the understory in the montane forest. All but one specimens of *B. phuyuhampatu* were found under mosses in the cloud forest around 2850 m a.s.l. (Fig. 5C). Male MUBI 14655 was found ~250 m from this site, under rocks and mosses under the riparian vegetation at the confluence of a small stream at 2795 m a.s.l. Two sympatric frogs, Gastrotheca cf. excubitor and *Psychrophrynella chirihampatu*, were found under rocks in disturbed habitats (i.e., along streams, landslides) but not in the cloud forest. Two additional amphibian species, *Bryophryne* sp. and B. cf. zonalis, were found along with G. cf. excubitor in the grasslands from 3100–3650 m a.s.l.

Both female paratopotypes had large eggs in their ovaries, indicative of terrestrial breeding and direct development: CORBIDI 18224 contained 15 eggs averaging 1.58 ± 0.05 mm in diameter (range 1.20–1.80 mm), while MUBI 16654 contained 16 eggs averaging 2.44 ± 0.03 mm in diameter (range 2.30–2.60 mm).

The type locality (and known distribution range) of the new species lies within the Área de Conservación Privada Ukumari Llaqta (Catenazzi and Ttito 2016), a protected area recognized by Peruvian environmental ministerial decree N° 301–2011-MINAM in December 2011. The upper puna and transitional habitats, as well as a narrow elevational band around the treeline are used for agriculture (potato cultivation), livestock (llamas grazing), fishing (exotic trout), and timber extraction. These land use patterns appear sustainable, and the grasslands at Patawasi (3350–3450 m a.s.l.) are in excellent conditions, with large bunchgrasses supporting large populations of *Bryophryne* sp. and B. cf. zonalis. There is little indication of human disturbance in the cloud forest, and the main use seems to be limited to trout fishing.

## Discussion

We assign the new species to *Bryophryne* on the basis of molecular data, shared meristic traits, general body shape and appearance, and overall similarity with the type species *B. cophites*, as well as with other species of *Bryophryne*. These frogs share robust bodies, short limbs, and usually lack a tympanic membrane and annulus (but they are present in *B. flammiventris* and *B. gymnotis*). Although no synapomorphy has been identified for external morphological characters, the geographic distribution within the Cusco region along with the molecular data support allocation of the new species to *Bryophryne*. Many recent descriptions within Holoadeninae have used molecular data as evidence supporting genus allocation (Catenazzi and Ttito 2016; Chaparro et al. 2015; Padial et al. 2012).

The diversity of high-elevation, small terrestrial-breeding frogs in the Department of Cusco has increased sharply over the past decade (Catenazzi and Ttito 2016; Catenazzi et al. 2015; Chaparro et al. 2007; Chaparro et al. 2015; De la Riva et al. 2008; Lehr and Catenazzi 2008; Lehr and Catenazzi 2009a; Lehr and Catenazzi 2009b; Lehr and Catenazzi 2010), mostly due to the addition of new species of *Bryophryne*. With the present description, three species of *Bryophryne* are known to occur in the region surrounding Abra (= mountain pass) Hualla Hualla and the upper Marcapata and adjacent valley (the other two being *Bryophryne* sp. and *B. zonalis*), equal to the number of species found around Abra Acjanaco (*B. cophites*, *B. hanssaueri*, and *B. nubilosus*) and Abra Málaga (*B. abramalagae*, *B. bustamantei*, and *B. gymnotis*). No other mountain pass has been surveyed as exhaustively as these three, and surveys in other mountain passes are likely to further increase the known diversity of the genus. Similarly to congeneric forms, *B. phuyuhampatu* appears to have a small geographic range, although it should be noted that the exact geographic and elevational range of forest dwelling species is poorly known at the moment. Two ecologically similar species occupy elevational ranges from 3195–3475 m a.s.l. (*B. hanssaueri*) and from 2340–3215 m a.s.l. (*B. nubilosus*) in the forests of the Kosñipata Valley (Catenazzi et al. 2013; Lehr and Catenazzi 2008; Lehr and Catenazzi 2009b), while *B. gymnotis* has been found from 3272–3354 m a.s.l. in the cloud forest near Abra Málaga.

*Bryophryne phuyuhampatu* occurs in a remote and protected area where no threats have been observed. Therefore, and according to the IUCN Red List criteria and categories (IUCN 2013), we propose to assign this species to the “Least Concern” category of the Red List. Although the amphibian pathogenic fungus *Batrachochytrium dendrobatidis* (Bd) has been reported in several frogs from the nearby region of Abra Hualla Hualla and Coline (~22 km S by airline from the type locality of *B. phuyuhampatu*) (Catenazzi et al. 2011), and is known to have caused the local extinction of many stream-breeding species in the montane forests of Manu NP (58 km NW of Quispillomayo), terrestrial-breeding frogs such as *Bryophryne* do not appear to be threatened by chytridiomycosis, and their populations have persisted during Bd epizootics (Catenazzi et al. 2011; Catenazzi et al. 2014; Warne et al. 2016). A survey of Bd infection in the nearby Japumayo Valley in 2015 found no infected frogs along an elevational transect from 2650–4600 m a.s.l. (Catenazzi and Ttito 2016). With the discovery of *Psychrophrynella chirihampatu* (Catenazzi and Ttito 2016) and *B. phuyuhampatu*, the Ukumary Llaqta protected area now boasts two endemic species not found in nationally protected areas, demonstrating the beneficial contribution of private protected areas to amphibian conservation (Catenazzi et al. 2015; Catenazzi and von May 2014; von May et al. 2008).

## Supplementary Material

XML Treatment for Bryophryne phuyuhampatu

## Appendix 1. Specimens examined

*Bryophryne bustamantei*: PERU: Cusco: Provincia La Convención: Abra de Málaga: MUSM 24537–38.

*Bryophryne cophites*: PERU: Cusco: Provincia de Paucartambo: Distrito Kosñipata: S slope Abra Acanaco, 14 km NNE Paucartambo, 3400 m: KU 138884 (holotype); N slope Abra Acanaco, 27 km NNE Paucartambo, 3450 m: KU 138885–908, 138911–5 (all paratypes); 2 km NE of Abra Acanaco, 3280 m: MHNG 2698.24, 5.5 km N of Abra Acanacu, 3523 m: MUSM 27895, Tres Cruces, 8.5 km N of Abra Acanaco, 3590 m: MUSM 20855–56, 26283–84, 26264, 26266–67, 26313, 26315, 27896, 30414–17, Pillco Grande, 3865 m, near border of Manu NP: CORBIDI 11919.

*Bryophryne gymnotis*: PERU: Cusco: Provincia de La Convención, Distrito de Huayopata: 1 km east of San Luis at elevations of 3272–3354 m: MUSM 24543 (holotype), MHNG 2710.28, 2710.29, MTD 46860–64, 47288, 47291–92, 47297, MUSM 24541–42, 24544–45, 24546–56, MVZ 258407–10 (all paratypes).

*Bryophryne hanssaueri*: PERU: Cusco: Provincia de Paucartambo, Distrito de Kosñipata: Acjanaco, Manu National Park, 3266 m elevation: MUSM 27567 (holotype); from near Acjanaco, Manu National Park at elevations of 3280–3430 m: MHNG 2698.25, MTD 46865–66, 46887–89, MUSM 24557, 27568–69, 27607–11, MVZ 258411–13 (all paratypes).

*Bryophryne nubilosus*: PERU: Cusco: Provincia de Paucartambo: Distrito de Kosñipata, 500 m NE of Esperanza, 2712 m: MUSM 26310 (holotype), MUSM 26311; near the type locality, 13°11'33.21"S, 71°35'25.17"W, 3065 m: MTD 47294; near Hito Pillahuata, 2600 m: MUSM 20970; Quebrada Toqoruyoc, 3097 m: MUSM 26312, MTD 47293; Esperanza, 2800 m: MHNSM 26316–17; 13°11'20.2''S, 71°35'07.3''W, 2900 m: MUSM 24539–40.

*Bryophryne* sp.: PERU: Cusco: Provincia de Quispicanchis: Distrito de Marcapata: Coline, 3672 m: MUSM 27571, 27573.

*Bryophryne zonalis*: PERU: Cusco: Provincia de Quispicanchis, Distrito de Marcapata, Kusillochayoc at 3129 m elevation: MUSM 27570 (holotype), MTD 46867, 46869–70, MUSM 27572, 27574–75, 27861, MVZ 258414 (all paratpyes); at Puente Coline, 3285 m elevation: MVZ 258415 (paratype).
